# Creatinine Clearance Is Associated with Toxicity from Molecularly Targeted Agents in Phase I Trials

**DOI:** 10.1159/000341152

**Published:** 2012-08-07

**Authors:** B. Basu, J. Vitfell-Pedersen, V. Moreno Garcia, M. Puglisi, A. Tjokrowidjaja, K. Shah, S. Malvankar, B. Anghan, J.S. de Bono, S.B. Kaye, L.R. Molife, U. Banerji

**Affiliations:** ^a^Drug Development Unit, Division of Clinical Studies, The Institute of Cancer Research/The Royal Marsden NHS Foundation Trust, Sutton, UK; ^b^Drug Development Unit, Division of Cancer Therapeutics, The Institute of Cancer Research/The Royal Marsden NHS Foundation Trust, Sutton, UK

**Keywords:** Cockcroft-Gault, Creatinine clearance, Molecularly targeted agent, Phase I trial, Toxicity

## Abstract

**Objectives:**

This study aimed to evaluate any correlations between baseline creatinine clearance and the development of grade 3/4 toxicities during treatment within oncology phase I trials of molecularly targeted agents where entry criteria mandate a serum creatinine of ≤1.5 × the upper limit of normal.

**Methods:**

Documented toxicity and creatinine clearance (calculated by the Cockcroft-Gault formula) from all patients treated with molecularly targeted agents in the context of phase I trials within our centre over a 5-year period were analyzed.

**Results:**

Data from 722 patients were analyzed; 116 (16%) developed at least one episode of grade 3/4 toxicity. Patients who developed a late-onset (>1 cycle) grade 3/4 toxicity had a lower creatinine clearance than those who did not (82.69 ml/min vs. 98.97 ml/min; p = < 0.001).

**Conclusion:**

Creatinine clearance (even when within normal limits) should be studied as a potential factor influencing late toxicities in the clinical trials of molecularly targeted anti-cancer drugs.

## Introduction

Phase I trials in oncology are designed to define a maximum tolerated dose and the safety and tolerability of new anti-cancer agents. Thus the evaluation of toxicity is fundamental to this objective [1,2]. The maximum tolerated dose is generally determined by the frequency of dose-limiting toxicity within the first cycle of treatment; dose-limiting toxicities are currently accepted as grade 3 (serious) or grade 4 (life-threatening) adverse events (AE) as determined by the National Cancer Institute's Common Terminology Criteria for Adverse Events and Common Toxicity Criteria evaluation. It is clearly important to identify factors that may predict the risk of developing significant side effects or fatality in these trials, given the likelihood that the patient may only derive modest clinical benefit from their participation in these studies [3,4,5].

Dosing of anti-cancer drugs in the era of cytotoxic therapy was traditionally individualised to body surface area (BSA) or glomerular filtration rate (GFR) [6]. Given the narrow therapeutic index of chemotherapy, the rationale for attempts at individualising dosing was to minimise inter-patient variability of drug exposure, with the expectation that reducing variability in pharmacokinetic profiles between patients would result in more predictable drug efficacy and toxicity [6]. However, BSA was shown to be poorly correlated with GFR [7] and subsequently, for agents that are primarily renally cleared, for example carboplatin, dosing strategies based upon accurate estimates of GFR rather than BSA were felt to improve inter-patient variability in PK [8]. Creatinine clearance (CrCL) – the volume of blood plasma that is cleared of creatinine per unit of time – is widely accepted as a simple measure of GFR. Alternative methods of CrCL calculation, for example by 24-hour urine collection, are subject to error from incomplete sample collection; therefore, it is more common to estimate GFR using formulae based upon a stable serum creatinine concentration [9]. The most widely used formula to estimate CrCL and GFR is the Cockcroft-Gault (CG) equation, which is based on serum creatinine, age, gender and weight [10].

Molecularly targeted agents (MTAs) are rapidly moving to the fore of onco-therapeutics. These are generally oral preparations, largely administered at fixed doses. Given their mechanism of action, it has been recommended that dose-finding for these agents be dependent on drug exposure resulting in target inhibition and biological effects in the tumour or surrogate tissue, with an absence of toxicity [11]. A nomogram quantifying the risk of treatment-related toxicity based on data from 578 cancer patients treated with MTAs alone or in combination in phase II trials at the Princess Margaret Hospital Drug Development Program, Toronto, identified baseline creatinine and BSA as predictors of a serious AE (attributable grade 3 or higher non-haematologic AE, or any grade 4 or higher haematological AE of any attribution) [12].

Currently, the inclusion criteria in oncology phase I trials specify, in most instances, a minimum renal function of serum creatinine level of ≤1.5 × the upper limit of normal or an estimated GFR of ≥60 ml/h. The rationale for this is 2-fold: (1) the metabolism/excretion of drugs is unknown in humans at this stage and (2) there should be a renal reserve in the event of drug induced renal toxicity.

In view of conflicting evidence in the literature over whether parameters such as GFR affect the inter-individual pharmacokinetic profiles of MTAs, we wished to establish whether clinical toxicity outcomes from MTAs could be influenced by variations in CrCL [13,14]. Therefore, the primary objective of this study was to determine whether CrCL was associated with the occurrence of grade 3 or grade 4 drug-related AEs in patients who had been administered novel MTAs in phase I clinical trials.

## Patients and Methods

### Patients

Data were analyzed from patients who were treated in a phase I trial of an MTA as monotherapy at the Drug Development Unit at the Institute of Cancer Research and Royal Marsden Hospital between January 2005 and December 2009. Patients treated with combinations of MTAs with cytotoxic agents were excluded. Inclusion and exclusion criteria across all studies were similar, including a baseline serum creatinine of ≤1.5 × the upper limit of normal. All patients consented to individual phase I trials approved by local research ethics committees. Patient case-report forms and clinical notes were used to collect baseline characteristics including age, height, weight and serum creatinine within 2 weeks prior to starting phase I trial treatment. The data were collected as part of an approved hospital audit studying in compliance with checking renal function in phase I clinical trials. A cycle of treatment was determined to be 21–35 days depending on the trial, and the first cycle of treatment was considered for evaluation of dose-limiting toxicities. Toxicity noted during the first cycle was called early toxicity and that noted after the first cycle was called late toxicity.

### Definition of Toxicity

Toxicity was defined using the National Cancer Institute's Common Terminology Criteria for Adverse Events and Common Toxicity Criteria evaluation for AEs, versions 2.0 or 3.0, depending on the version in use at the time of the study. Data collected included all grade 3 and grade 4 toxicities felt to be possibly, probably or likely to be related to the investigational drug.

Serum creatinine was measure by the modified Jaffe method using Beckman Coulter DxC.

### Estimation of GFR

The CG formula was used to calculate an estimate of GFR (CrCL) in ml/min:

(140 – age) × weight (kg) × *k*/SCr

where *k* = 1.23 for males and 1.04 for females, SCr = serum creatinine (µmol/l).

### Statistics

CrCL was categorized into 3 groups: low <60 ml/min, intermediate 60–120 ml/min and high >120 ml/min. The χ^2^ test was used to analyze differences in the incidence of toxicity between these groups. A Student t test was used to compare CrCL means between patients with toxicity and patients without toxicity. A binary multivariate regression model with forward selection (likelihood ratio) was performed with clinical variables of weight, height, gender and age to determine their relationship with the development of toxicity. A Kruskal-Wallis analysis of variance was used to test for differences in the median CrCL for patients with no toxicity, early toxicity (cycle 1) or late toxicity (later than cycle 1). All tests were 2-tailed and p ≤ 0.05 was considered statistically significant. Statistical analysis was performed using an SPSS program (version 17.0; SPSS, Chicago, Ill., USA).

## Results

### Patient and Trial Characteristics

A total of 722 patients who participated in 45 different phase I trials of MTAs were included in the analysis. Of the cohort studied, 100% of the patients entered into the trials met the eligibility criteria based on renal function assessments. Their baseline characteristics are shown in table 1 and targets of MTA with which they were treated are shown in table 2. All 722 patients included in this analysis had recorded creatinine values at the start of a trial. In 657 of them, additional information including weight, age and sex was available in order to calculate CrCl by the CG formula.

### Toxicities and Creatinine Clearance

**Fig. 1:**
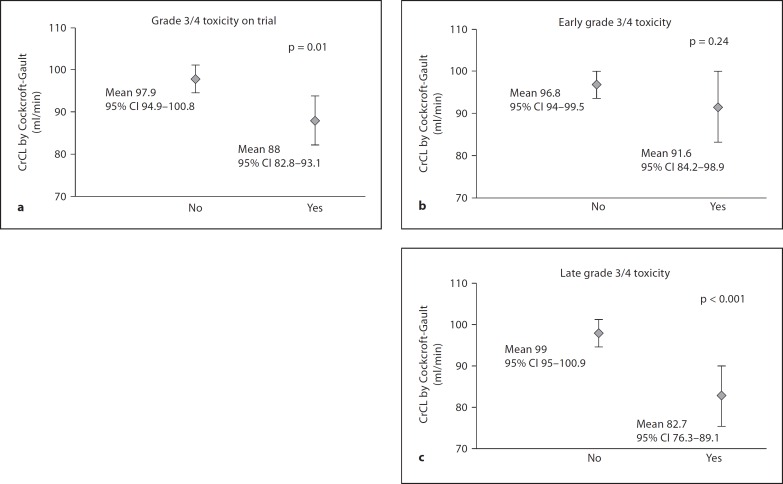
Patients who develop toxicities within phase I trials have lower baseline CrCL values than those who do not. Presence (YES) or absence (NO) of grade 3/4 toxicities plotted against CrCL (mean ± 95% CI) throughout phase I trial (**a**), during cycle 1 (**b**) and occurring beyond cycle 1 (**c**). There were no significant differences in CrCL for patients who developed grade 3 or 4 toxicites within the first cycle of the study; however, patients who developed serious toxicity beyond cycle 1 had lower baseline CrCL values than patients who did not develop late toxicities.

One-hundred-and-sixteen patients (16%) experienced at least one episode of grade 3 or grade 4 toxicity, and grade 5 toxic death was observed for 4 patients during the study period. In 72 (10%) patients the toxicity appeared during cycle 1 and in 44 (6%) patients the first toxicity appeared beyond cycle 1. For the entire cohort, patients developing grade 3 or grade 4 toxicity during their phase I trial showed lower mean baseline CrCL values (88 ml/min) compared to patients who did not develop these throughout the trial (97.9 ml/min) (p = 0.01) (fig. 1a). This analysis showed a discrepancy between early versus late toxicity for patients with different renal function. There was no significant difference in CrCL for patients developing grade 3/4 toxicities in cycle 1 (CrCL 91.6 ml/min in patients with early toxicity vs. 96.8 ml/min in patients without toxicity in cycle 1; p = 0.24) (fig. 1b). However, patients who developed a late (beyond cycle 1) grade 3 or grade 4 toxicity had a lower mean CrCL than those who did not (82.69 ml/min vs. 98.9 ml/min; p < 0.001) (fig. 1c).

**Fig. 2:**
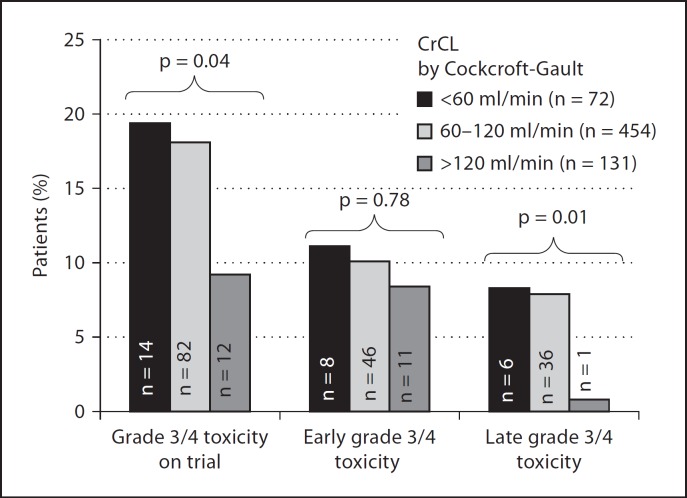
Lower CrCL values are associated with a greater incidence of grade 3/4 toxicity in phase I studies of MTAs in cancer patients. Patients within phase I studies of MTAs were subdivided according to their CrCL: low <60 ml/min, intermediate 60–120 ml/min and high >120 ml/min. Patients with a low CrCl had a significantly higher grade 3/4 toxicity compared to those who had a high CrCl. This difference in toxicities between low and high CrCl was noted after the first cycle of treatment.

Frequency of grade 3/4 toxicity at any point in the trial was similar for patients with low baseline CrCL and intermediate CrCL (19 and 18% of patients, respectively). However, patients with a high CrCL showed a significantly lower incidence of grade 3 and grade 4 toxicity (9%) (p = 0.04) (fig. 2). Again, this was as a result of a reduced incidence of late toxicity (after cycle 1) for those patients with a high baseline CrCL (>120 ml/min) since only 1 of 131 patients (0.8%) developed late toxicity compared to 8.3 and 7.9% of patients with low (<60 ml/min) and intermediate (60–120 ml/min) CrCL, respectively (p = 0.01) (fig. 2). No differences were observed in the rates of grade 3 and grade 4 toxicity across the differing levels of renal function during the first cycle of trial treatment. In a multivariate logistic regression model for toxicity, CrCL was significantly associated with the incidence of grade 3 and 4 toxicity (OR = 0.99, 95% CI 0.98–0.99, p = 0.028); however, factors contributing to calculation of the CrCL by CG, namely, age, gender and weight, showed no significant independent association with the development of severe toxicity.

## Discussion

This single-centre retrospective study of patients treated in phase I trials of MTAs shows that even with rigorous trial selection criteria for renal function, a lower CrCL, as calculated by the CG method, is associated with a higher incidence of serious toxicity. Of interest, this was more relevant for toxicities developing outside the conventional dose-limiting toxicity-defining period in cycle 1. Traditionally, the recommended phase II dose for these drugs is established from AEs observed in the first cycle of treatment; however, it is becoming increasingly clear that toxicities emerging beyond this period should also be considered, especially taking into account the chronic dosing schedules for many MTAs, often beyond the customary 6 (i.e. 21- to 28-day cycles) [15]. The identification of risk factors that may predict the development of serious toxicity of MTAs could potentially help minimise the risk for patients undergoing phase I trials and this is especially important given the low overall probability of significant clinical benefit at this stage. A recent analysis of risk factors for MTA treatment related AEs in early-phase cancer clinical trials did not evaluate CrCL [5].

From our dataset, it appears that, despite standard phase I trial eligibility criteria, there is an adequate range of renal function within this study population to identify differential susceptibility to toxicity. We hypothesize that patients with a spectrum of renal function even within the ‘normal’ range, may, on treatment over time, display subtle changes in the accumulation of renally-excreted metabolites, alterations in plasma protein-binding and drug distribution that are sufficient to impact on the tolerability of MTAs. We believe that, in this analysis, the heterogeneity of agents across many different trials with differing mechanisms of action and elimination may strengthen the argument that renal function is independently associated with toxicities on these treatments.

Despite the low contribution of renal clearance to drug exposure of many MTAs currently in clinical practice, there are some indications of differential safety profiles in patients with varying renal function. In one retrospective study in metastatic renal cancer patients, analyzing the safety and efficacy of MTAs (sunitinib, temsirolimus, everolimus or bevacizumab) with respect to GFR, patients with CrCL ≤60 ml/min showed larger median increases in blood pressure on sunitinib and bevacizumab therapy, an increased incidence of thyroid dysfunction with sunitinib and a greater incidence of rash and dose interruptions with mTOR inhibitors than did patients with normal renal function [14]. In contrast, another study has shown a significant increase in imatinib exposure in patients with mild (CrCL 40–59 ml/min) to moderate (CrCL 20–39 ml/min) renal impairment, without any association with an increased incidence of toxicities [13]. It is possible that, for other MTAs, a similar phenomenon of increased exposure with decreasing renal function may translate to an increased incidence of serious toxicity.

The finding that CrCL is associated with an increased risk of late (>cycle 1) grade 3 and grade 4 toxicity in the setting of normal renal function in phase I trials of MTAs is of interest and of potential relevance in the design of phase I studies as well as in their assessment. The emergence of late grade 3–4 toxicities from MTAs beyond cycle 1 lends weight to the argument for incorporating the evaluation of late toxicities into recommended phase II dose assessments, and could potentially aid the formulation of strategies for dose modifications when designing phase II studies. Initially, the association of CrCL in patients with ‘normal’ renal function or varying degrees of renal dysfunction and late grade 3–4 toxicity could be studied in the maximum tolerated dose expansion stages of phase I or phase II studies. If early-phase data for a given MTA demonstrated a higher incidence of late grade 3 and 4 toxicities in the context of differing renal function, associations between the number of dose de-escalations or interruptions and CrCL could then be studied prospectively in larger phase II or phase III clinical trials.

## Figures and Tables

**Table 1:** Baseline characteristics of 722 patients

Characteristic	
Age, years	59 (18–85)
Weight, kg	74 (39–135)
Height, cm	166 (l45–196)
Serum creatinine, μmol/l	74 (38–175)
Creatinine clearance ml/min	
(Cockcroft-Gault)	89 (26–269)
Gender	
Male	372 (51)
Female	350 (49)
Ethnicity	
White	668 (92.5)
Asian	18 (2.5)
Afro-American	20 (2.8)
Unknown	16 (2.2)
Co-morbidities	318 (44)
Hypertension	123 (17)
Diabetes mellitus	36 (5)
Vascular disease	56 (8)
Lung disease/COPD	52 (7)
Liver disease	1 (1)
ECOG	
0	216 (30)
1	460 (64)
2	46 (6)
Grade 3/4 toxicity	
First cycle	72 (10)
After first cycle	44 (6)
No	606 (84)

Data are expressed as medians (range) or number of patients (percentages).

**Table 2:** Phase I trial agents

Target	Number of trials	Number of patients (% of total)	Early toxicity (% of target)	Late toxicity (% of target)
Cell cycle and apoptosis	6	53 (7)	5 (9)	3 (6)
Chromatin remodelling	8	97 (13)	13 (13)	6 (6)
Anti-sense	2	3 (1)	0	1 (33)
Cytoplasmic signalling protein	9	123 (17)	18 (15)	9 (7)
DNA repair	3	71 (10)	3 (4)	4 (6)
Growth factor receptors	14	206 (29)	21 (10)	13 (6)
Oncolytic virus	5	41 (6)	6 (15)	1 (2)
Protein folding and degradation	4	29 (4)	3 (10)	2 (7)
Anti-angiogenic/vascular	4	24 (3)	1 (4)	2 (8)
Other	4	75 (10)	2 (3)	3 (4)
